# Retention and marginal bone loss in attachment systems for implant retained overdentures

**DOI:** 10.6026/973206300210485

**Published:** 2025-03-31

**Authors:** Surabhi Duggal, Sahba Hassan, Khadija Anwaar, Yogesh Garg, Shailesh Jain

**Affiliations:** 1 Department of Prosthodontics and Crown & Bridge, School of Dental Sciences, Sharda University, Greater Noida, Uttar Pradesh-201310, India; 2 Department of Prosthodontics and Crown & Bridge, DJ College of Dental Sciences and Research, Modinagar, Uttar Pradesh-201204, India; 3 Department of Public Health Dentistry, JCD Dental College, Sirsa, Haryana-125055, India; 4 Department of Prosthodontics and Crown & Bridge, Bhojia Dental College, Baddi, Himachal Pradesh- 173205, India

**Keywords:** Dental implantation, dental prosthesis, marginal bone loss, ball attachment

## Abstract

Comparison of retention and marginal bone loss of ball, bar and locator attachment systems for implant retained overdentures it is of interest. Hence, related data were collected from 75 patients. Retention was highest for the bar system, while the locator and ball systems exhibited moderate and significant reductions. The bar attachment system outperformed ball and locator systems in retention, marginal bone preservation and patient satisfaction, making it the most effective option for implant-retained overdentures.

## Background:

Implant-retained overdentures have revolutionized prosthodontics by offering improved function, comfort and esthetics for edentulous patients. Out of the several types of attachment systems available for attaching overdentures to implants, ball, bar and locator attachments form the most common list [1]. These systems vary considerably depending on designed architecture, biomechanical properties and clinical performance primarily concerning with implant survival and marginal bone alterations around implants in the medium and long term [2]. Retention is patient oriented since it defines the stability of the prosthesis during function, while marginal bone loss is implant success and longevity oriented. While long term care dentures appear to be relatively economical in comparison with various implant supported prostheses, they provide little satisfaction to the patients in terms of stability and masticatory efficiency particularly in the mandibular region [3]. Each of them has peculiarities that define such clinical behavior of the given attachment system. The ball attachment system is highly preferred, as it is relatively cheaper and easy to implement [4]. It is made of a head of a small metal sphere partially embedded in the denture base and a counter-part is a conical cavity into which the head of the stud fits. This system is comparatively very simple to construct and to fix and for this reason, many clinicians opt for it. Still, its retention capabilities may degrade over time as we see in wear of the retentive elements [5]. Furthermore, ball attachments can apply rotatory loads on the implants resulting in marginal bone resorption in certain situations. Bar attachment system is on the contrary characterized by a metal bar where one end has implants and the overdenture attaches to the other end of the bar through clips or other retentive features [6]. This design also affords superior primary stability and initial retention, particularly for patients who have lost much of their osseous support or those for whom a more substantial modicum of prosthetic support is deemed necessary [7]. They spread the occlusal forces evenly on the implants and this should reduce stress on the particular implant contributing to prevention of bone loss. Nevertheless, this system is more sophisticated in design and fabrication since it entails comprehensive planning as compared to designs with higher initial costs. Locator attachments are relatively new in implant dentistry [8]. These are concealed, very stable and made to allow for changes in the angulation of the implants. Silica Locator Systems offer excellent retention characteristics as a consequence of their geometries and can be used in most try-in situations where composite retention needs to be optimized [9]. A comparison of three types of attachment systems has revealed that different research results concern bone loss - bar systems in most cases are least affected due to force distribution. Ball and locator systems may cause higher bone loss, if forces are not balanced and sustained in the centre [10]. Therefore, it is of interest to report that the bar attachment system demonstrated the most favourable outcomes in terms of retention, marginal bone preservation and patient satisfaction.

## Methodology:

This prospective observational study was conducted at reputed Dental College of North India and data were collected from 75 patients. All participants were >40 years of age, with no systemic conditions that could affect bone metabolism or healing, such as diabetes or osteoporosis. They had adequate bone volume to support implant placement without the need for grafting procedures. Written informed consent was obtained from all participants.

## Data collection:

Participants were randomly divided into three equal groups of 25 patients each, based on the type of attachment system to be used:

[1] Group A: Patients received ball attachment systems.

[2] Group B: Patients received bar attachment systems.

[3] Group C: Patients received locator attachment systems.

Each participant received two implants in the anterior mandible using a standardized surgical protocol. The implants were placed in the inter-foraminal region, ensuring parallel alignment to optimize retention and minimize stress on the attachment systems. A 60 day osseointegration period was allowed before connection of attachment systems. For all participants, custom-made mandibular overdentures were made. As for the dentures, these were fabricated to meet compatibility with the attachment system allocated and suitability to the perfect fitting/functioning. The colour of the all prostheses was heat-cured acrylic resin with the use of metal reinforcements for high strength. Measurement assessments were made at pre-intervention (n = 94; immediately after attachment placement) and post intervention (at three months (n = 67), six months (n = 57) and twelve months (n = 48). Retention was quantified by applying means of using a digital force gauge, which was needed to remove the overdenture off the implants. To familiarise the experiment with standard conditions, the measurements were performed in similar conditions. The radio-graphic assessment of the marginal bone levels was also done using standardized digital radiographs. Standardized periapical radiographs were made at baseline and at the time of each follow-up examination to document graft resorption and implant integration around the implants. They were taken with the help of imaging software and have the accuracy of 0.01 mm. Patients' opinions were also surveyed using a standardised postal questionnaire completed before and after using the attachment system for at least two weeks; providing data related to comfort, ease of use and overall satisfaction with the attachment system.

## Statistical analysis:

Data were analyzed using SPSS v26. Descriptive statistics were used to summarize demographic and baseline characteristics. A one-way A NOVA was performed to compare retention and marginal bone loss between the three groups. Post-hoc tests were conducted to identify significant differences between pairs of groups. A p-value of <0.05 was considered statistically significant.

## Results:

Data were collected from 75 patients; bar attachment system consistently exhibited the highest retention values throughout the 12-month study period, starting at 35.2 ± 3.0 N at baseline and decreasing to 30.8 ± 3.0 N by 12 months. The locator attachment system demonstrated moderate retention, beginning at 28.4 ± 2.5 N and reducing to 24.2 ± 2.7 N over the same period. The ball attachment system showed the lowest retention values, with a significant decline from 20.5 ± 2.1 N at baseline to 15.6 ± 2.0 N at 12 months (Table 1). The marginal bone loss results show that the bar attachment system consistently exhibited the least bone loss, with values increasing from 0.30 ± 0.04 mm at 3 months to 0.85 ± 0.08 mm at 12 months. The locator attachment system demonstrated moderate bone loss, ranging from 0.40 ± 0.06 mm at 3 months to 1.05 ± 0.09 mm at 12 months. The ball attachment system experienced the highest bone loss, starting at 0.45 ± 0.05 mm at 3 months and reaching 1.25 ± 0.10 mm by 12 months (Table 2). The patient satisfaction results highlight that the bar attachment system received the highest scores across all parameters, including comfort (8.9 ± 0.7), ease of use (8.5 ± 0.8) and overall satisfaction (9.0 ± 0.8). The locator attachment system also performed well, particularly in ease of use (8.6 ± 0.7) and achieved an overall satisfaction score of 8.4 ± 0.7. The ball attachment system, while providing moderate comfort (7.5 ± 0.8) and ease of use (7.8 ± 0.7), had the lowest overall satisfaction score of 7.6 ± 0.9. (Table 3). The retention percentage reduction over time indicates that the bar attachment system demonstrated the least decline in retention, with a reduction of 4.83% at 3 months, 9.09% at 6 months and 12.50% at 12 months. The locator attachment system showed a moderate reduction, starting at 5.28% at 3 months and increasing to 14.79% by 12 months. The ball attachment system experienced the highest retention loss, with a significant reduction of 8.78% at 3 months, 17.07% at 6 months and 23.90% at 12 months (Table 4).

## Discussion:

The findings of this study provide valuable insights into the comparative performance of ball, bar and locator attachment systems for implant-retained overdentures, particularly concerning retention, marginal bone loss and patient satisfaction. Stability and functionality of implant-retained overdentures are closely related with the rate of retention. For the bar attachment system, retention was higher throughout the 12-month study period and minimal loss was recorded throughout the study period (12.5% only at 12 months) [11]. This may be due to its structure that spreads distribution of forces on several implants and a firm fixing point to the prosthesis [12]. Again, this study pointed out that the locator abutment provided better retention than the ball attachment because of its low profiled biocompatible resilient inserts which continuously offer the abutment a lasting connection to the implant. That is why it can be concluded that the configuration with the ball attachment system demonstrated the greatest decline in the retention rate, dropping from 76.1 percent to 52.2 percent after 12 months and showing the highest wear rate that may require more frequent maintenance or replacement of the attachment system elements [13]. Marginal bone loss is the most important parameter in implant success and sustainability. The study shows slight reduction in marginal bone loss and it was least for the bar attachment system (0.85 ± 0.08 mm) at 12 months of prosthetic loading, probably owing to the inherent characteristics of the bar attachment system to dispense the vertical occlusal load uniformly throughout and thereby avoiding the concentrated stress on individual implants. The locator system had a moderate bone resorption of 1.05 ± 0.09 mm which could be attributed by the focussing of forces round the implants because of the highly resilient attachment procedure of the locator system. These results support prior research indicating that societal programs with more favourable force distribution tend to help maintain additional peri-implant bone. The bar attachment received the highest rating concerning the overall satisfaction (9. 0± 0.8) arising from its excellent retention and stability. Patients were also comfortable; this could be because of even distribution of the load with least movement of the overdenture. The locator system was considered to be more user-friendly and less conspicuous or obtrusive than the pointer compared with the pointer system due to less hand manipulation by the patient.

## Conclusion:

The bar attachment system is the most effective option for implant-retained overdentures, demonstrating superior retention, minimal marginal bone loss with the highest patient satisfaction scores over a 12-month period. Its ability to evenly distribute occlusal forces across implants contributes to its success in preserving peri-implant bone and ensuring long-term stability. The locator attachment system, while slightly less effective in retention and bone preservation, offers a practical and user-friendly solution for patients seeking moderate cost and ease of maintenance.

## Figures and Tables

**Table 1:** Retention values (in Newton's)

**Time Interval**	**Ball Attachment (Group A)**	**Bar Attachment (Group B)**	**Locator Attachment (Group C)**
Baseline	20.5 ± 2.1 N	35.2 ± 3.0 N	28.4 ± 2.5 N
3 Months	18.7 ± 2.3 N	33.5 ± 3.1 N	26.9 ± 2.6 N
6 Months	17.0 ± 2.2 N	32.0 ± 3.2 N	25.5 ± 2.8 N
12 Months	15.6 ± 2.0 N	30.8 ± 3.0 N	24.2 ± 2.7 N

**Table 2:** Marginal bone loss (in Millimeters)

**Time Interval**	**Ball Attachment (Group A)**	**Bar Attachment (Group B)**	**Locator Attachment (Group C)**
3 Months	0.45 ± 0.05 mm	0.30 ± 0.04 mm	0.40 ± 0.06 mm
6 Months	0.80 ± 0.07 mm	0.55 ± 0.05 mm	0.70 ± 0.08 mm
12 Months	1.25 ± 0.10 mm	0.85 ± 0.08 mm	1.05 ± 0.09 mm

**Table 3:** Patient satisfaction scores (Scale: 1-10)

**Parameter**	**Ball Attachment (Group A)**	**Bar Attachment (Group B)**	**Locator Attachment (Group C)**
Comfort	7.5 ± 0.8	8.9 ± 0.7	8.2 ± 0.6
Ease of Use	7.8 ± 0.7	8.5 ± 0.8	8.6 ± 0.7
Overall Satisfaction	7.6 ± 0.9	9.0 ± 0.8	8.4 ± 0.7

**Table 4:** Retention percentage reduction over time

**Time Interval**	**Ball Attachment (Group A)**	**Bar Attachment (Group B)**	**Locator Attachment (Group C)**
3 Months	8.78%	4.83%	5.28%
6 Months	17.07%	9.09%	10.21%
12 Months	23.90%	12.50%	14.79%
